# 2,8-Dibromo-4,10-dichloro-6*H*,12*H*-5,11-methano­dibenzo[*b*,*f*][1,5]diazo­cine

**DOI:** 10.1107/S1600536808026226

**Published:** 2008-08-20

**Authors:** Kai-Xian Zhu, Donald C. Craig, Andrew C. Try

**Affiliations:** a Department of Chemistry and Biomolecular Sciences, Building F7B, Macquarie University, Sydney, NSW 2109, Australia; b School of Chemistry, University of New South Wales, Sydney, NSW 2052, Australia

## Abstract

The title compound, C_15_H_10_Br_2_Cl_2_N_2_, a 2,8-dibromo-4,10-dichloro Tröger’s base analogue derived from 4-bromo-2-chloro­aniline, has a dihedral angle of 110.9 (10)° between the two aryl rings, the largest yet measured for a simple dibenzo analogue.

## Related literature

For related literature on the synthesis and crystal structures of dihalogenated Tröger’s base analogues, see: Jensen & Wärnmark (2001 ▶); Faroughi *et al.* (2006*a* ▶, 2007*a* ▶,*b* ▶). For Tröger’s base analogues substituted with multiple electron-withdrawing groups, see: Faroughi *et al.* (2006*b* ▶); Bhuiyan *et al.* (2006 ▶, 2007 ▶); Vande Velde *et al.* (2008 ▶). For reactions of halogenated Tröger’s base analogues, see: Jensen *et al.* (2002 ▶); Hof *et al.* (2005 ▶). For literature on racemization of Tröger’s base analogues and the effect of substituents *ortho* to the diazo­cine N atoms, see: Lenev *et al.* (2006 ▶).
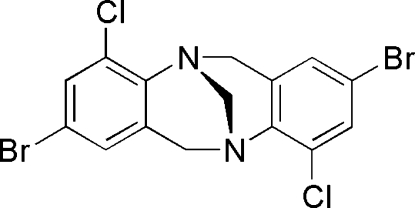


## Experimental

### 

#### Crystal data

C_15_H_10_Br_2_Cl_2_N_2_
*M* *_r_* = 449.0
Orthorhombic, 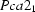

*a* = 7.910 (2) Å
*b* = 12.601 (3) Å
*c* = 15.230 (4) Å
*V* = 1518.0 (7) Å^3^
*Z* = 4
Mo *K*α radiation
μ = 5.64 mm^−1^
*T* = 294 K
0.30 × 0.12 × 0.07 mm

#### Data collection

Enraf–Nonius CAD-4 diffractometer
Absorption correction: analytical (de Meulenaer & Tompa, 1965 ▶) *T*_min_ = 0.52, *T*_max_ = 0.69
1394 measured reflections
1394 independent reflections
1028 reflections with *I* > 2σ(*I*)
1 standard reflection frequency: 30 min intensity decay: none

#### Refinement

*R*[*F* ^2^ > 2σ(*F* ^2^)] = 0.056
*wR*(*F* ^2^) = 0.061
*S* = 1.61
1394 reflections
189 parameters
H-atom parameters constrained
Δρ_max_ = 0.98 e Å^−3^
Δρ_min_ = −1.02 e Å^−3^
Absolute structure: Flack (1983 ▶)
Flack parameter: 0.09 (2)

### 

Data collection: *CAD-4* (Schagen *et al.*, 1989 ▶); cell refinement: *CAD-4*; data reduction: local program; program(s) used to solve structure: *SIR92* (Altomare *et al.*, 1994 ▶); program(s) used to refine structure: *RAELS* (Rae, 1996 ▶); molecular graphics: *ORTEPII* (Johnson, 1976 ▶); software used to prepare material for publication: local programs.

## Supplementary Material

Crystal structure: contains datablocks global, I. DOI: 10.1107/S1600536808026226/tk2290sup1.cif

Structure factors: contains datablocks I. DOI: 10.1107/S1600536808026226/tk2290Isup2.hkl

Additional supplementary materials:  crystallographic information; 3D view; checkCIF report

## Figures and Tables

**Fig. 1.**
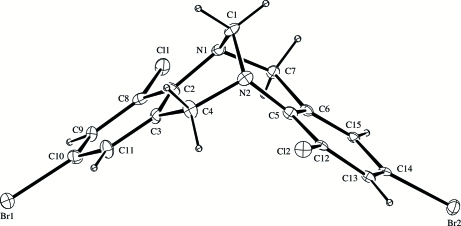
ORTEPII (Johnson, 1976) plot of (I), with ellipsoids at the 10% probability level. H atoms are drawn as spheres of arbitrary radius.

**Fig. 2.**
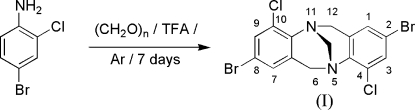
Synthetic scheme for the synthesis of (I) showing the numbering system used in naming the compound.

## supplementary crystallographic information

### Comment

Tröger's base analogues bearing electron-withdrawing groups were long thought to be difficult, if not impossible, to prepare. However, the synthesis of dihalogenated (Jensen & Wärnmark, 2001), octafluoro (Vande Velde *et al.*, 2008) and tetranitro (Bhuiyan *et al.*, 2007) Tröger's base analogues highlight the possiblities that now exist in terms of incorporating electron-withdrawing groups on the starting anilines. The synthetic utility of halogen-substituted Tröger's base analogue has been demonstrated with their conversion to alkyne- (Jensen & Wärnmark, 2001; Jensen *et al.*, 2002) and functionalized phenyl- (Hof *et al.*, 2005) substituted analogues, among others. It is noteworthy that crystal structures of several other 2,4,8,10-tetrasubstituted Tröger's base analogues exhibit large dihedral angles that are close to that in (I). Tröger's base analogues are known to undergo racemization in acidic solution, however the presence of a substituent at the *ortho*-position, relative to the bridge nitrogen atoms, has been shown to increase the racemization barrier (Lenev *et al.*, 2006).

The molecular structure of (I) is shown in Fig. 1 and it was prepared as outlined in Fig. 2.

### Experimental

4-Bromo-2-chloroaniline (1 g, 4.84 mmol) and paraformaldehyde (232 mg, 7.74 mmol) were added to an ice-cold solution of trifluoroacetic acid (10 ml). The reaction mixture was then stirred in dark at room temperature for 7 days under an atmosphere of argon. The ice-cold reaction mixture was basified by the dropwise addition of a mixture of ammonia (28%, 20 ml) and water (40 ml), followed by the additon of a saturated sodium hydrogen carbonate solution (20 ml). The resultant mixture was then extracted with dichloromethane (3 *x* 20 ml) and the combined organic layers were washed with brine (40 ml), dried over anhydrous sodium sulfate, filtered and evaporated to dryness. The crude product was chromatographed (silica gel, dichloromethane:hexane 8:2) to afford 2,8-dibromo-4,10-dichloro-6*H*,12*H*-5,11-methanodibenzo [*b*,*f*][1,5]diazocine (I) (613 mg, 56%) as a white solid and as a racemic mixture: m.p. 471–472 K; ^1^H NMR (400 MHz, CDCl_3_) δ 4.21–4.33 (4*H*, m), 4.55 (2*H*, d, *J* 17.3 Hz), 7.04 (2*H*, d, *J* 2.1 Hz), 7.41 (2*H*, d, *J* 2.1 Hz); ^13^C NMR (100 MHz, CDCl_3_) δ 54.37, 67.32, 117.15, 128.49, 130.17, 131.02, 131.71, 142.33. Analysis found: C 40.46; H 2.22; N 6.46; C_15_H_10_Br_2_Cl_2_N_2_ requires C 40.13; H 2.25; N 6.24. Single crystals were obtained from slow evaporation from dichloromethane solution of (I).

### Refinement

Hydrogen atoms were included in positions calculated each cycle (C—H = 1.0 Å), and were assigned thermal parameters equal to their bonded atom. The maximum and minimum electron density peaks were located 0.73 and 1.20Å from the Cl2 and Br1 atoms, respectively.

### Crystal data

**Table:**

C_15_H_10_Br_2_Cl_2_N_2_	*D*_x_ = 1.96 Mg m^−^^3^
*M**_r_* = 449.0	Melting point: 471 K
Orthorhombic, *P**c**a*2_1_	Mo *K*α radiation λ = 0.71073 Å
Hall symbol: P 2c -2ac	Cell parameters from 11 reflections
*a* = 7.910 (2) Å	θ = 10–11º
*b* = 12.601 (3) Å	µ = 5.64 mm^−^^1^
*c* = 15.230 (4) Å	*T* = 294 K
*V* = 1518.0 (7) Å^3^	Prism, colourless
*Z* = 4	0.30 × 0.12 × 0.07 mm
*F*_000_ = 872.0	

### Data collection

**Table:**

Enraf–Nonius CAD-4 diffractometer	θ_max_ = 25º
ω–2θ scans	*h* = 0→9
Absorption correction: analyticalde Meulenaer & Tompa (1965)	*k* = 0→14
*T*_min_ = 0.52, *T*_max_ = 0.69	*l* = −18→0
1394 measured reflections	1 standard reflections
1394 independent reflections	every 30 min
1028 reflections with *I* > 2σ(*I*)	intensity decay: none

### Refinement

**Table:**

Refinement on *F*	*w* = 1/[σ^2^(*F*) + 0.0004*F*^2^]
*R*[*F*^2^ > 2σ(*F*^2^)] = 0.056	(Δ/σ)_max_ = 0.002
*wR*(*F*^2^) = 0.061	Δρ_max_ = 0.98 e Å^−^^3^
*S* = 1.61	Δρ_min_ = −1.02 e Å^−^^3^
1394 reflections	Extinction correction: none
189 parameters	Absolute structure: Flack (1983), 0 Friedel pairs
H-atom parameters constrained	Flack parameter: 0.09 (2)

### Fractional atomic coordinates and isotropic or equivalent isotropic displacement parameters (Å^2^)

**Table:**

	*x*	*y*	*z*	*U*_iso_*/*U*_eq_	
Br1	0.8061 (2)	0.6867 (1)	0.3991 (1)	0.0585 (5)	
Br2	0.2324 (2)	0.0800 (1)	0.0428 (2)	0.0527 (5)	
Cl1	0.9770 (5)	0.5167 (4)	0.0779 (3)	0.057 (1)	
Cl2	0.5654 (5)	0.0565 (3)	0.3603 (3)	0.052 (1)	
N1	0.9460 (14)	0.3064 (10)	0.1658 (8)	0.041 (3)	
N2	0.8319 (15)	0.1842 (9)	0.2739 (8)	0.044 (3)	
C1	0.9798 (19)	0.2123 (12)	0.2198 (10)	0.044 (4)	
C2	0.8998 (18)	0.3929 (12)	0.2193 (10)	0.043 (4)	
C3	0.8445 (18)	0.3790 (11)	0.3054 (8)	0.041 (4)	
C4	0.817 (2)	0.2693 (12)	0.3414 (8)	0.049 (4)	
C5	0.6842 (19)	0.1696 (11)	0.2226 (9)	0.039 (4)	
C6	0.6740 (19)	0.2086 (11)	0.1360 (9)	0.036 (4)	
C7	0.8206 (17)	0.2753 (12)	0.0978 (9)	0.040 (4)	
C8	0.9155 (17)	0.4984 (13)	0.1855 (9)	0.043 (4)	
C9	0.8825 (19)	0.5861 (12)	0.2397 (11)	0.048 (4)	
C10	0.839 (2)	0.5684 (13)	0.3247 (11)	0.050 (4)	
C11	0.818 (2)	0.4666 (13)	0.3570 (10)	0.056 (4)	
C12	0.5565 (17)	0.1082 (10)	0.2538 (8)	0.031 (3)	
C13	0.4158 (17)	0.0854 (10)	0.2036 (8)	0.035 (3)	
C14	0.4117 (17)	0.1221 (11)	0.1168 (9)	0.040 (4)	
C15	0.5433 (19)	0.1819 (10)	0.0853 (9)	0.034 (3)	
H1C1	1.0780	0.2273	0.2593	0.044	
H2C1	1.0078	0.1513	0.1804	0.044	
H1C4	0.7008	0.2660	0.3674	0.049	
H2C4	0.9027	0.2559	0.3883	0.049	
H1C7	0.7726	0.3410	0.0708	0.040	
H2C7	0.8794	0.2326	0.0517	0.040	
HC9	0.8906	0.6600	0.2162	0.048	
HC11	0.7825	0.4567	0.4195	0.056	
HC13	0.3194	0.0438	0.2286	0.035	
HC15	0.5411	0.2061	0.0228	0.034	

### Atomic displacement parameters (Å^2^)

**Table:**

	*U*^11^	*U*^22^	*U*^33^	*U*^12^	*U*^13^	*U*^23^
Br1	0.060 (1)	0.060 (1)	0.0549 (9)	−0.0011 (8)	−0.001 (1)	−0.0138 (9)
Br2	0.0470 (9)	0.0650 (9)	0.0461 (8)	−0.0118 (8)	−0.0026 (9)	−0.0091 (9)
Cl1	0.061 (3)	0.066 (3)	0.044 (2)	−0.019 (2)	0.010 (2)	0.000 (2)
Cl2	0.063 (3)	0.056 (2)	0.037 (2)	0.000 (2)	0.006 (2)	0.018 (2)
N1	0.029 (7)	0.058 (8)	0.037 (7)	0.001 (6)	0.002 (6)	−0.003 (6)
N2	0.046 (7)	0.048 (7)	0.038 (7)	0.004 (6)	−0.009 (6)	0.020 (6)
C1	0.029 (9)	0.067 (9)	0.036 (8)	0.005 (8)	−0.016 (7)	0.012 (8)
C2	0.051 (9)	0.043 (9)	0.034 (8)	0.009 (8)	0.020 (8)	−0.006 (7)
C3	0.045 (9)	0.059 (9)	0.019 (8)	−0.012 (8)	−0.004 (7)	−0.005 (7)
C4	0.083 (9)	0.053 (8)	0.013 (7)	0.004 (9)	−0.006 (7)	0.007 (7)
C5	0.048 (9)	0.043 (9)	0.026 (7)	0.000 (7)	0.013 (7)	0.007 (7)
C6	0.039 (8)	0.042 (8)	0.026 (8)	0.010 (8)	0.002 (6)	−0.006 (7)
C7	0.037 (8)	0.052 (9)	0.030 (8)	−0.007 (8)	0.007 (7)	−0.002 (7)
C8	0.029 (8)	0.062 (9)	0.039 (9)	−0.002 (8)	−0.002 (7)	0.004 (9)
C9	0.047 (9)	0.038 (9)	0.059 (9)	−0.010 (8)	−0.001 (8)	−0.007 (8)
C10	0.047 (9)	0.057 (9)	0.046 (9)	−0.005 (8)	0.017 (8)	−0.007 (8)
C11	0.085 (9)	0.052 (9)	0.029 (8)	−0.011 (9)	0.009 (9)	−0.007 (8)
C12	0.037 (8)	0.032 (8)	0.024 (7)	0.009 (7)	0.010 (6)	−0.002 (6)
C13	0.046 (9)	0.043 (9)	0.016 (6)	0.014 (8)	0.001 (6)	0.000 (7)
C14	0.029 (8)	0.039 (8)	0.052 (9)	0.006 (7)	0.010 (7)	−0.015 (8)
C15	0.034 (8)	0.039 (8)	0.027 (7)	0.004 (7)	0.009 (7)	−0.004 (7)

### Geometric parameters (Å, °)

**Table:**

Br1—C10	1.890 (15)	C4—H2C4	1.000
Br2—C14	1.888 (14)	C5—C6	1.410 (18)
Cl1—C8	1.724 (14)	C5—C12	1.358 (18)
Cl2—C12	1.750 (13)	C6—C7	1.546 (20)
N1—C1	1.468 (18)	C6—C15	1.333 (19)
N1—C2	1.409 (17)	C7—H1C7	1.000
N1—C7	1.487 (18)	C7—H2C7	1.000
N2—C1	1.474 (19)	C8—C9	1.404 (20)
N2—C4	1.491 (18)	C9—C10	1.357 (19)
N2—C5	1.417 (18)	C9—HC9	1.000
C1—H1C1	1.000	C10—C11	1.385 (21)
C1—H2C1	1.000	C11—HC11	1.000
C2—C3	1.394 (18)	C12—C13	1.381 (17)
C2—C8	1.431 (20)	C13—C14	1.402 (18)
C3—C4	1.503 (20)	C13—HC13	1.000
C3—C11	1.371 (20)	C14—C15	1.371 (19)
C4—H1C4	1.000	C15—HC15	1.000
			
C1—N1—C2	110.4 (12)	N1—C7—C6	112.5 (11)
C1—N1—C7	107.4 (11)	N1—C7—H1C7	108.7
C2—N1—C7	115.7 (11)	N1—C7—H2C7	108.7
C1—N2—C4	106.0 (11)	C6—C7—H1C7	108.7
C1—N2—C5	112.2 (11)	C6—C7—H2C7	108.7
C4—N2—C5	114.1 (12)	H1C7—C7—H2C7	109.5
N1—C1—N2	111.3 (11)	Cl1—C8—C2	119.3 (11)
N1—C1—H1C1	109.0	Cl1—C8—C9	120.4 (12)
N1—C1—H2C1	109.0	C2—C8—C9	120.2 (12)
N2—C1—H1C1	109.0	C8—C9—C10	118.6 (15)
N2—C1—H2C1	109.0	C8—C9—HC9	120.7
H1C1—C1—H2C1	109.5	C10—C9—HC9	120.7
N1—C2—C3	121.9 (14)	Br1—C10—C9	118.5 (13)
N1—C2—C8	119.2 (12)	Br1—C10—C11	120.0 (11)
C3—C2—C8	118.8 (13)	C9—C10—C11	121.4 (15)
C2—C3—C4	120.3 (13)	C3—C11—C10	121.6 (14)
C2—C3—C11	119.1 (14)	C3—C11—HC11	119.2
C4—C3—C11	120.6 (12)	C10—C11—HC11	119.2
N2—C4—C3	113.5 (10)	Cl2—C12—C5	120.5 (12)
N2—C4—H1C4	108.4	Cl2—C12—C13	117.9 (10)
N2—C4—H2C4	108.4	C5—C12—C13	121.6 (13)
C3—C4—H1C4	108.4	C12—C13—C14	118.2 (13)
C3—C4—H2C4	108.4	C12—C13—HC13	120.9
H1C4—C4—H2C4	109.5	C14—C13—HC13	120.9
N2—C5—C6	121.2 (13)	Br2—C14—C13	119.2 (11)
N2—C5—C12	119.6 (13)	Br2—C14—C15	121.0 (11)
C6—C5—C12	118.9 (15)	C13—C14—C15	119.6 (13)
C5—C6—C7	119.8 (13)	C6—C15—C14	121.6 (13)
C5—C6—C15	119.9 (14)	C6—C15—HC15	119.2
C7—C6—C15	120.1 (12)	C14—C15—HC15	119.2
			
C2—N1—C1—N2	57.5 (15)	C2—C3—C11—C10	2.3 (24)
C2—N1—C1—H1C1	−62.8	C2—C3—C11—HC11	−177.7
C2—N1—C1—H2C1	177.8	C4—C3—C11—C10	−178.6 (16)
C7—N1—C1—N2	−69.4 (15)	C4—C3—C11—HC11	1.4
C7—N1—C1—H1C1	170.3	N2—C5—C6—C7	−4.4 (20)
C7—N1—C1—H2C1	50.8	N2—C5—C6—C15	171.2 (13)
C1—N1—C2—C3	−18.2 (18)	C12—C5—C6—C7	−177.9 (12)
C1—N1—C2—C8	159.5 (13)	C12—C5—C6—C15	−2.3 (20)
C7—N1—C2—C3	103.9 (16)	N2—C5—C12—Cl2	5.4 (18)
C7—N1—C2—C8	−78.3 (17)	N2—C5—C12—C13	−175.2 (12)
C1—N1—C7—C6	44.6 (15)	C6—C5—C12—Cl2	179.1 (10)
C1—N1—C7—H1C7	165.1	C6—C5—C12—C13	−1.6 (20)
C1—N1—C7—H2C7	−75.8	C5—C6—C7—N1	−10.2 (17)
C2—N1—C7—C6	−79.1 (15)	C5—C6—C7—H1C7	−130.7
C2—N1—C7—H1C7	41.3	C5—C6—C7—H2C7	110.2
C2—N1—C7—H2C7	160.4	C15—C6—C7—N1	174.2 (13)
C4—N2—C1—N1	−69.8 (13)	C15—C6—C7—H1C7	53.8
C4—N2—C1—H1C1	50.5	C15—C6—C7—H2C7	−65.3
C4—N2—C1—H2C1	169.9	C5—C6—C15—C14	3.9 (21)
C5—N2—C1—N1	55.3 (16)	C5—C6—C15—HC15	−176.1
C5—N2—C1—H1C1	175.6	C7—C6—C15—C14	179.5 (12)
C5—N2—C1—H2C1	−65.0	C7—C6—C15—HC15	−0.5
C1—N2—C4—C3	42.4 (15)	Cl1—C8—C9—C10	−178.1 (12)
C1—N2—C4—H1C4	163.0	Cl1—C8—C9—HC9	1.9
C1—N2—C4—H2C4	−78.2	C2—C8—C9—C10	1.4 (23)
C5—N2—C4—C3	−81.5 (16)	C2—C8—C9—HC9	−178.6
C5—N2—C4—H1C4	39.1	C8—C9—C10—Br1	176.8 (11)
C5—N2—C4—H2C4	157.9	C8—C9—C10—C11	−3.7 (25)
C1—N2—C5—C6	−17.3 (19)	HC9—C9—C10—Br1	−3.2
C1—N2—C5—C12	156.2 (13)	HC9—C9—C10—C11	176.3
C4—N2—C5—C6	103.2 (14)	Br1—C10—C11—C3	−178.6 (12)
C4—N2—C5—C12	−83.3 (16)	Br1—C10—C11—HC11	1.4
N1—C2—C3—C4	−5.9 (21)	C9—C10—C11—C3	1.9 (26)
N1—C2—C3—C11	173.3 (14)	C9—C10—C11—HC11	−178.1
C8—C2—C3—C4	176.4 (14)	Cl2—C12—C13—C14	−176.9 (10)
C8—C2—C3—C11	−4.4 (21)	Cl2—C12—C13—HC13	3.1
N1—C2—C8—Cl1	4.3 (19)	C5—C12—C13—C14	3.7 (19)
N1—C2—C8—C9	−175.2 (13)	C5—C12—C13—HC13	−176.3
C3—C2—C8—Cl1	−177.9 (11)	C12—C13—C14—Br2	173.5 (9)
C3—C2—C8—C9	2.6 (21)	C12—C13—C14—C15	−2.1 (18)
C2—C3—C4—N2	−7.5 (20)	HC13—C13—C14—Br2	−6.5
C2—C3—C4—H1C4	−128.1	HC13—C13—C14—C15	177.9
C2—C3—C4—H2C4	113.1	Br2—C14—C15—C6	−177.3 (11)
C11—C3—C4—N2	173.3 (14)	Br2—C14—C15—HC15	2.7
C11—C3—C4—H1C4	52.7	C13—C14—C15—C6	−1.7 (20)
C11—C3—C4—H2C4	−66.1	C13—C14—C15—HC15	178.3
